# Enzyme Biosensor Based on 3D-Printed Flow-Through Reactor Modified with Thiacalixarene-Functionalized Oligo (Lactic Acids)

**DOI:** 10.3390/bios15020077

**Published:** 2025-01-29

**Authors:** Dmitry Stoikov, Dominika Kappo, Alexey Ivanov, Vladimir Gorbachuk, Olga Mostovaya, Pavel Padnya, Ivan Stoikov, Gennady Evtugyn

**Affiliations:** 1 Alexander Butlerov Institute of Chemistry, Kazan Federal University, 18 Kremlevskaya Street, Kazan 420008, Russia; dmistojkov@kpfu.ru (D.S.); dkappo@kpfu.ru (D.K.); olga.mostovaya@mail.ru (O.M.); padnya.ksu@gmail.com (P.P.); ivan.stoikov@mail.ru (I.S.); 2 Analytical Chemistry Department, Chemical Technology Institute, Ural Federal University, 19 Mira Street, Ekaterinburg 620002, Russia

**Keywords:** flow-through analysis, chronoamperometry, electrochemical biosensor, replaceable reactor, uricase, thiacalixarene-functionalized oligo (lactic acids)

## Abstract

Electrochemical enzyme biosensors are extensively utilized in clinical analysis and environmental monitoring, yet achieving effective enzyme immobilization while maintaining high activity remains a challenge. In this work, we developed a flow-through enzyme biosensor system using a 3D-printed flow-through electrochemical cell fabricated from commercially available poly (lactic acid). After modification with thiacalixarene-functionalized oligo (lactic acids) (OLAs), the material enabled efficient immobilization of uricase on the inner surface of a replaceable reactor of the cell. Swelling and hydrolytic stability of OLAs in *cone*, *partial cone*, and *1,3-alternate* conformations were studied, with *1,3-alernate* conformation demonstrating superior stability and enzyme immobilization performance. The use of OLAs enhanced immobilization efficiency by over 30% and protected the reactor from swelling, hydrolytic degradation, and enzyme loss. The biosensor was validated for amperometric uric acid determination, with a screen-printed carbon electrode modified with carbon black and Prussian Blue. This modification reduced the cathodic potential for uric acid detection to –0.05 V. The biosensor exhibited a linear detection range of 10 nM to 30 μM with a detection limit of 7 nM, and it performed effectively in artificial urine and synthetic blood plasma. The novel cell design, featuring easy assembly and low-cost replaceable parts, makes this biosensor a promising candidate for routine clinical analysis and other practical applications.

## 1. Introduction

The current progress in medicine, biotechnology, the food industry, and environmental protection necessitates the development of highly sensitive methods for detecting various compounds, such as ecotoxicants, disease biomarkers, and drugs [1]. Classical chromatographic and mass spectrometric methods meet the required sensitivity standards but present several significant drawbacks, including high equipment and consumable costs, labor-intensive procedures, maintenance complexities, and the need for highly qualified personnel to operate the instruments [2]. Electrochemical sensors offer a promising alternative due to their low cost, ease of use, potential for miniaturization, and capacity for rapid analysis in online [3] and point-of-care testing modes [4] while maintaining high sensitivity and selectivity [5]. Additionally, the capability of performing electrochemical detection in a flow-through mode enables further automation of the measurement process and facilitates the analysis of a large number of samples in continuous flow conditions [6].

The development of enzyme-based biosensors, which utilize enzymes as biorecognition elements, is particularly appealing due to their ability to achieve high specificity and sensitivity for target analytes [7]. One such analyte of interest is uric acid [8,9,10] (7,9-dihydro-1*H*-purine-2,6,8(3*H*)-trione), the final metabolite of purines in humans [11]. An elevated plasma uric acid concentration above 404 μM indicates hyperuricemia [12] and may serve as a marker for conditions such as gout [13], anemia [14], psoriasis [15], and pneumonia [16], among others. Therefore, the determination of uric acid levels in biological fluids holds significant potential for the early diagnosis of these diseases. To determine uric acid, enzyme sensors based on uricase, an enzyme from the class of oxidoreductases, are used. This is a class of enzymes that catalyze oxidation/reduction reactions by facilitating the transfer of electrons from reducing agents to oxidizers [17].

The use of free enzymes in sensor compositions poses challenges due to their low stability and difficulty in reusability during sensor operation [18]. To immobilize enzymes on sensor surfaces, various methods are available, including adsorption, electrostatic interactions, and covalent binding [19]. A critical challenge in enzyme sensor development is identifying an optimal enzyme immobilization strategy that ensures maximum enzyme activity is retained.

The design of most enzyme sensors described in the literature involves immobilizing the biocomponent on the surface of the transducer, typically the working electrode [20,21,22]. However, these sensors are not well-suited for flow-through assays because the immobilized enzyme can be washed away from the electrode surface during operation in a carrier stream. Furthermore, the optimal conditions for enzyme functionality, such as pH and temperature, are often within a narrow range [23], which imposes strict requirements for modifying the working electrode. These challenges can be addressed by spatially separating the transducer and the enzyme, with the latter immobilized on the surface of an independent reactor.

In enzyme sensors, amperometry is one of the most commonly used methods for signal detection [24,25]. Amperometric sensors detect changes in the electric current that occur due to oxidation and reduction reactions involving the analyte [26]. These current changes are proportional to the analyte concentration and are measured using an electrode set at a specific potential to enhance electron transfer.

3D printing technology is currently actively used in the field of electrochemical analysis for the development of electrochemical devices [27,28,29]. In a previous work, we proposed a series of amperometric flow-through biosensor systems featuring a 3D-printed, replaceable enzyme reactor made of poly(lactic acid) (PLA) [30,31,32]. The physical separation of the reactor from the working screen-printed carbon electrode (SPCE) enhanced system stability under flow conditions and minimized adverse effects of the reagents used for enzyme immobilization and SPCE modification.

PLA is a thermoplastic polymer widely used in various industries due to its unique properties [33]. PLA exhibits excellent barrier and mechanical characteristics [34], making it suitable for both prototyping and the production of functional components using 3D printing technology [35]. The key feature of PLA is its biodegradability [36], which makes it an attractive material for the production of packaging, textiles, and medical products. Moreover, the biocompatibility of PLA [37] fostered an optimal environment for enzymatic reactions involving diverse enzymes [38,39] and their substrates.

Recent studies have focused on developing nanomaterials [40] and polymer coatings [41] as carrier matrices for enzyme immobilization to improve enzyme stability and activity. In this context, modifying the surface of PLA reactors with functionalized macrocyclic compounds is particularly promising. Functionalization of oligo- and poly(lactic acids) with cyclophanes has been shown to enhance their affinity for various low- and high-molecular-weight substrates [42], while also affecting self-assembly properties and thermal stability [43]. Additionally, the conformation of thiacalix[4]arenes significantly influences the analytical performance of electrochemical sensors [44].

Despite the exceptional selectivity of enzyme-based biosensors toward the analyte, which stems from the inherent specificity of enzymes, the intrinsic redox activity of the enzyme’s active site is insufficient to generate a robust response signal [45]. The sensitivity of biosensor systems to analytes is also affected by the modification of the working electrode. Redox-active compounds [46], composites [47], and polymeric materials [48] are widely used as electrode modifiers. These materials act as electron transfer mediators, improving electron exchange conditions at the electrode-solution interface. Among these, Prussian blue (PB), a synthetic pigment with the formula $Fe_{4}^{III}[Fe^{II}(CN{)}_{6}{]}_{3}$, holds promise as a working electrode modifier due to its reversible redox properties, making it an efficient mediator in electrochemical processes [49]. Various methods have been developed for electrode surface modification with PB, including electrochemical deposition [50], chemical deposition [51], and the incorporation of nanostructured PB [52]. Combining PB with carbon nanostructures, polymers, or enzyme systems produces synergistic effects that enhance sensor performance [53].

In this study, thiacalixarene-functionalized oligo (lactic acids) in different conformations (*cone*, *partial cone*, and *1,3-alternate*) were employed as modifiers for PLA in the fabrication of a 3D-printed flow-through electrochemical cell-based enzyme biosensor reactor. The swelling behavior and hydrolytic degradation of the thiacalixarene-functionalized oligo (lactic acids) were investigated. Prussian blue was used to modify the working electrode in the development of the biosensor. The resulting flow-through biosensor system was utilized for the determination of hydrogen peroxide and uric acid.

## 2. Materials and Methods

### 2.1. Reagents

Carbon black (CB) N220 was purchased from Cabot (Ravenna, Italy). The suspension of oxidized CB was prepared according to our previously developed methodology [54].

**Figure 1:**
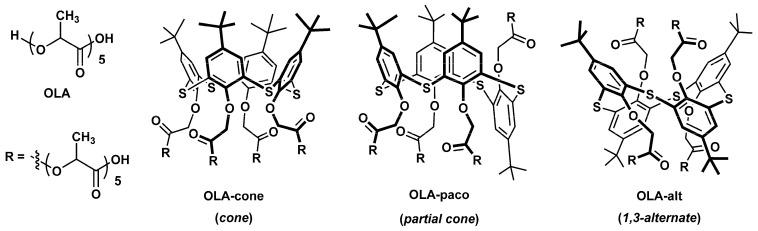
Chemical structures of oligo (lactic acids) without macrocyclic core (OLA) and functionalized by *p*-*tert*-butylthiacalix[4]arene in *cone* (OLA-cone), *partial cone* (OLA-paco), and *1,3-alternate* (OLA-alt) conformations [55,56].

Uricase from Candida sp. (EC 1.7.3.3, lyophilized powder, 4.5 U/mg solid), uric acid, PLA, *N*-(3-dimethylaminopropyl)-*N*′-ethylcarbodiimide chloride (EDC), and *N*-hydroxysuccinimide (NHS) were purchased from Sigma-Aldrich (St. Louis, MO, USA). Oligo (lactic acids) without macrocyclic core (OLA) and modified with *p*-*tert*-butylthiacalix[4]arenes in *cone* (OLA-cone), *partial cone* (OLA-paco), and *1,3-alternate* (OLA-alt) conformations (Figure 1) were synthesized according to our previously developed methodology [55,56].

The working solutions were prepared using deionized Millipore-Q water (Simplicity^®^ water purification system, Merck-Millipore, Molsheim, France). Artificial urine consisted of urea (416 mM), KCl (21 mM), KH_2_PO_4_ (20 mM), NH_4_Cl (18 mM), CaCl_2_ (10 mM), creatinine (9 mM), MgCl_2_ (6 mM), Na_2_SO_4_ (6 mM), and potassium citrate (2 mM) [57]. Synthetic blood plasma contained NaCl (2 mM), NaHCO_3_ (0.2 mM), *L*-tryptophan (0.21 mM), *L*-tyrosine (0.2 mM), *L*-alanine (0.2 mM), *L*-aspartic acid (22 μM), *DL*-lysine (5 μM), *L*-arginine (5 μM), *L*-methionine (4 μM), *L*-phenylalanine (4 μM), *L*-histidine (3.5 μM), *L*-glycine (3.5 μM), and *L*-cysteine (1.3 μM) [58].

### 2.2. Modification of Screen-Printed Carbon Electrode (SPCE)

SPCEs were manufactured with the DEK 248 printer (DEK, London, UK) according to our previously developed methodology [54]. Modification of the working electrode was performed by drop-casting 1 μL of a suspension containing CB at a concentration of 0.66 mg·mL⁻^1^ in propylene carbonate. Following this, 5 μL each of 0.1 M K₃[Fe(CN)₆] and 0.1 M FeCl₃ in a solution of 0.1 M HCl with 0.1 M KCl were applied dropwise onto the CB layer and incubated for 10 min. The electrode was then rinsed with 0.1 M HCl and dried at 100 °C for 60 min.

### 2.3. Flow-Through Cell Manufacture

**Figure 2:**
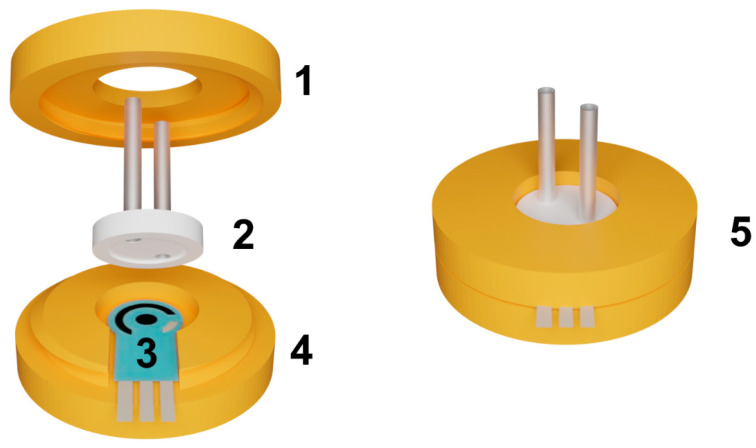
The assembly of the flow-through cell. (**1**)–upper part of the cell with an internal thread; (**2**)–replaceable reactor; (**3**)–SPCE, (**4**)–bottom part of the cell with an external thread; (**5**)–assembled cell.

The flow-through cell was fabricated using a Wanhao Duplicator 9/300 3D printer (Wanhao, Jinhua, China) equipped with a 0.3 mm nozzle extruder. Commercially available PLA filaments (Bestfilament, Moscow, Russia) with a diameter of 1.75 mm were used as the printing material due to their ease of 3D printing, biodegradability, cost-effectiveness, and satisfactory processing precision. The manufacturer-specified characteristics of the PLA plastic are as follows: density—1.23–1.25 g/cm^3^, water absorption—0.2–0.4%, melting temperature: 155–170 °C, glass transition temperature—60 °C; resistance to temperatures up to 70 °C; high mechanical strength; flexibility and elasticity. The printing parameters included a layer thickness of 0.1 mm, a printing speed of 700 mm·s⁻^1^, and a printing temperature of 220 °C. The reactor consisted of two parts that were assembled using screws and flat washers. Once assembled, the flow-through cell measured 3.5 × 3.5 × 1.2 cm (Figure 2).

The flow-through cell body consisted of two components: the bottom part with an external thread and the top part with an internal thread. The top part featured a round hole designed to accommodate capillaries entering the reactor. The outer diameter of the reactor was larger than the diameter of the hole in the top part, allowing the top part to exert pressure on the reactor when tightened. The reactor had an inner radius of 9 mm and a depth of 0.3 mm. This design ensured a secure seal by pressing the reactor tightly against the electrode, creating an airtight system without the need for additional sealing elements. The sealing of the system plays a critical role in ensuring its stability. To verify the sealing, a flow rate 10 times higher than the operating flow rate (exceeding the operating pressure) is passed through the cell after assembly. The sealing is deemed sufficient if no air bubbles are observed in the liquid exiting the cell and no liquid leakage is detected.

### 2.4. Enzyme Immobilization and Signal Measurement

The enzyme was immobilized on the inner side of the replaceable reactor (Figure 2(2)), which was modified with one of the oligo (lactic acids). To immobilize the enzyme, the reactor was secured in upside down position. A 10 µL aliquot of thiacalixarene-functionalized oligo (lactic acid) (OLA-cone, OLA-paco, or OLA-alt) in acetone was drop-cast onto the inner walls of the reactor and allowed to dry completely. Subsequently, 5 µL of 100 mM EDC was mixed with 5 µL of 400 mM NHS and applied to the inner walls of the reactor. After a 10 min incubation, the reactor was rinsed with deionized water. Next, 15 µL of a uricase solution (5 U) were applied to the reactor’s inner notch and left to dry at ambient temperature for 60 min. Immobilization was achieved through carbodiimide binding, involving the terminal carboxylic groups of PLA and the amino groups of the enzyme molecules. The reactor was then gently rinsed with a working buffer followed by deionized water. After assembling, the cell working solutions were pumped through the system using a Model 100 syringe pump (ALS, Tokyo, Japan). The cathodic reduction current of hydrogen peroxide, generated in the enzymatic reaction, was recorded in chronoamperometric mode using a BioStat multichannel potentiostat (ESA Bioscience Inc., Chelmsford, MA, USA). Cyclic voltammograms were recorded using a CHI 660E potentiostat (CH Instruments Inc., Austin, TX, USA).

### 2.5. Hydrolytic Degradation and Swelling of Oligo (Lactic Acids)

The hydrolytic degradation and swelling behavior of thiacalixarene-functionalized OLA samples were assessed using a gravimetric method [59,60,61]. Experiments were conducted in three separate 500 mL glass containers, each filled with 200 mL of buffer solutions of varying pH: pH 4.0 (50 mM potassium phthalate), pH 6.9 (50 mM potassium dihydrophosphate), and pH 9.2 (10 mM sodium borate). Thin OLA films (200 mg) were prepared on pre-weighed glass plates (2 × 6 cm) using a Shimadzu AUW 120-D analytical balance (Shimadzu Corporation, Kyoto, Japan). The initial weight of the samples was recorded as w_0_. The prepared plates with the OLA films were placed vertically in the buffer solutions for incubation at room temperature (20 ± 2 °C) under the following conditions: 24 h (pH = 4.0), 24 and 168 h (pH = 6.9), and 4 h (pH = 9.2).

After incubation, the plates were removed, and excess water was gently blotted away using a fiber cloth. The wet weight of the samples (w_w_) was measured, after which the samples were dried in an oven at 100 °C for 24 h to achieve a constant weight. The dry weight (w_d_) was then recorded. To ensure reproducibility, all experiments were performed in triplicate.

The degree of swelling was calculated by comparing the wet weight (w_w_) with the dry weight (w_d_) using Equation (1):

(1) $$D\mathrm{egree}\;\mathrm{of}\;\mathrm{swelling}\;\left(\%\right)=\frac{w_{w}-w_{d}}{w_{w}}\;\times \;100$$

The percentage of weight loss due to hydrolytic degradation was determined by comparing the initial weight (w_0_) to the dry weight (w_d_) using Equation (2):

(2) $$W\mathrm{eight}\;l\mathrm{oss}\;\left(\%\right)=\frac{w_{0}-w_{d}}{w_{0}}\;\times \;100$$

## 3. Results and Discussion

### 3.1. Design of 3D-Printed Flow-Through Cell

The flow-through electrochemical cell utilized in this study was designed with a focus on miniaturization and ease of assembly (Figure 2). Unlike most commercially available flow-through devices that require additional fasteners for assembly, this cell features a simplified design that closes with a single rotary motion due to the threaded connections between the upper and bottom parts. The pressure exerted by the upper part against the reactor ensures a secure seal within the internal space between the electrode and the reactor. This design eliminates the need for additional sealing elements, further simplifying the construction.

**Scheme 1:**
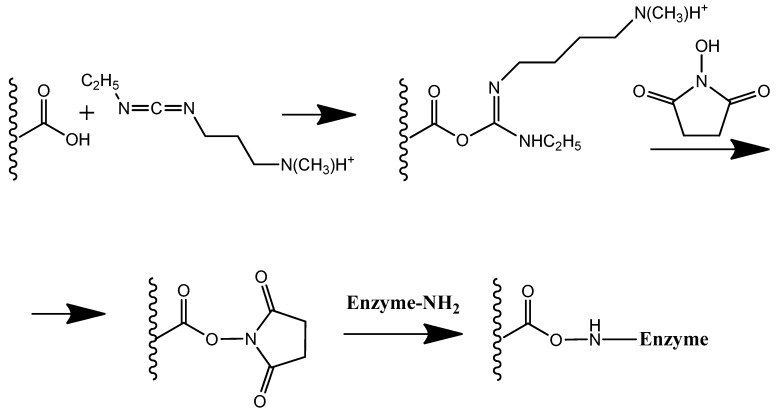
Carbodiimide binding of enzyme to PLA with terminal carboxyl groups.

Both the inner reactor and the cell body were 3D-printed using PLA, a material that enables modification of the inner surface of the reactor with enzymes via carbodiimide cross-linking with terminal carboxyl groups (Scheme 1). In earlier studies, we introduced the concept of physically separating the biocomponent from the electrode transducer by employing a 3D-printed PLA reactor [30]. In the present work, the reactor surface was pre-modified with thiacalixarene-functionalized oligo (lactic acids) to enhance its functional properties.

### 3.2. Swelling and Hydrolytic Degradation of Thiacalixarene-Functionalized Oligo (Lactic Acids) Used to Modify the Flow-Through Electrochemical Cell Reactor Chamber

PLAs are widely utilized in applications ranging from surgical materials and drug delivery systems to everyday consumer products, primarily due to their non-toxicity and biosorbability [61]. A key advantage of PLAs is their unique combination of biocompatibility and biodegradability [62]. The biodegradation of these materials is critically influenced by the swelling stage, which determines the rate of hydrolytic degradation [63].

In this study, thiacalixarene-functionalized oligo (lactic acids) (OLA-cone, OLA-paco, and OLA-alt) were proposed as modifiers for the flow-through reactor cell. These polyfunctional and branched structures have the potential to enhance enzyme immobilization efficiency while simultaneously protecting the reactor from excessive swelling and hydrolytic degradation. To assess the impact of the macrocyclic core and its conformation, the swelling and hydrolytic degradation of films composed of OLA-cone, OLA-paco, and OLA-alt were evaluated in three buffer solutions with pH values of 4.0 (after 24 h), 6.9 (after 24 and 168 h), and 9.2 (after 4 h) (Table 1). For comparison, an oligo (lactic acid) (OLA) sample containing an equivalent amount of lactic acid residues was included in this study.

As a rule, oligo- and poly(lactic acids) undergo degradation in both alkaline and acidic media due to the hydrolysis of ester bonds. This degradation is particularly pronounced in alkaline environments, where it is irreversible. The modification of oligo (lactic acid) with thiacalixarene derivatives was found to significantly reduce both weight loss and the degree of swelling (Table 1). The observed effect followed a pattern among the stereoisomers in the order of OLA-cone < OLA-paco < OLA-alt. This trend can be attributed to a decrease in the amphiphilic nature of the conformations in this series. Typically, thiacalixarenes in the *cone* conformation exhibit greater water solubility due to their amphiphilic structure, wherein the substituents are located on one side of the macrocyclic platform [64]. Conversely, the highly branched *1,3-alternate* stereoisomer results in a more hydrophobic overall structure. Consequently, the stability of thiacalixarene-functionalized oligo (lactic acids) is influenced by pH, following the trend pH 9.2 < pH 4.0 < pH 6.9. The highest stability is observed at neutral pH, which is close to the physiological pH of blood and optimal conditions for enzyme activity.

### 3.3. Registration of Hydrogen Peroxide Using a Prussian Blue-Based Sensor

Based on the properties of thiacalixarene-functionalized oligo (lactic acids), these compounds were selected to modify the flow-through electrochemical cell reactor. This modification aimed to reduce potential swelling and provide a suitable surface for enzyme immobilization. However, utilizing an enzymatic reaction in electrochemical studies necessitates further modification of the working electrode. Since oxidase enzymes were used, a sensitive modifying coating for hydrogen peroxide detection was investigated.

Hydrogen peroxide is a crucial analyte in diverse fields, including biomedical diagnostics [65], environmental monitoring [66], and food processing [67]. It is also a byproduct of various enzymatic reactions [68]. Electroanalytical methods, particularly amperometric sensors, are widely employed for hydrogen peroxide detection due to their simplicity, cost-effectiveness, and compatibility with miniaturization [69]. Prussian Blue (PB) has emerged as a promising material for modifying such sensors, offering enhanced analytical performance compared to unmodified electrodes, owing to its unique catalytic, electrochemical, and selective properties [70]. Prussian Blue is a well-established transducer for hydrogen peroxide detection, with its electrochemical behavior and analytical application comprehensively reviewed in [71].

In this study, PB was synthesized directly on SPCEs pre-modified with CB. To prepare the PB-modified electrode, 5 μL each of 0.1 M K_3_[Fe(CN)_6_] and 0.1 M FeCl_3_ solutions in 0.1 M HCl were sequentially applied dropwise to the working surface of the electrode. The modified electrode was incubated for 10 min, washed with 0.1 M HCl, and dried for 60 min at 100 °C. Cyclic voltammograms were recorded after the modification exhibited a pair of redox peaks within the potential range of 0.075 V to 0.125 V, which can be attributed to the reaction described in Equation (3) [71].

(3) $$Fe_{4}^{III}[Fe^{II}(CN{)}_{6}{]}_{3}+4e^{-}+4K^{+}↔K_{4}Fe_{4}^{II}[Fe^{II}(CN{)}_{6}{]}_{3}$$

**Figure 3:**
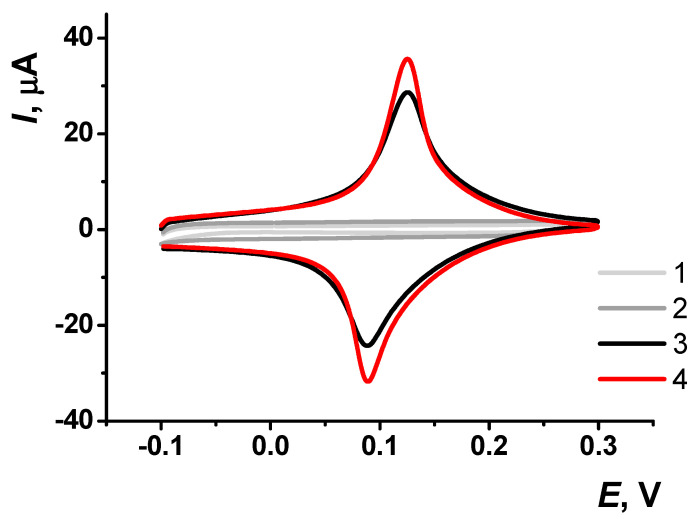
Cyclic voltammograms obtained on screen-printed carbon electrodes (SPCE): (1) unmodified SPCE, (2) SPCE modified with carbon black (CB), (3) SPCE modified with Prussian Blue (PB), and (4) SPCE modified with a pre-application of CB followed by PB. Voltammetric measurements were carried out in 0.01 M phosphate buffer solution + 0.1 KCl, pH = 7.0. Scan rate 0.1 V∙s^−1^.

**Figure 4:**
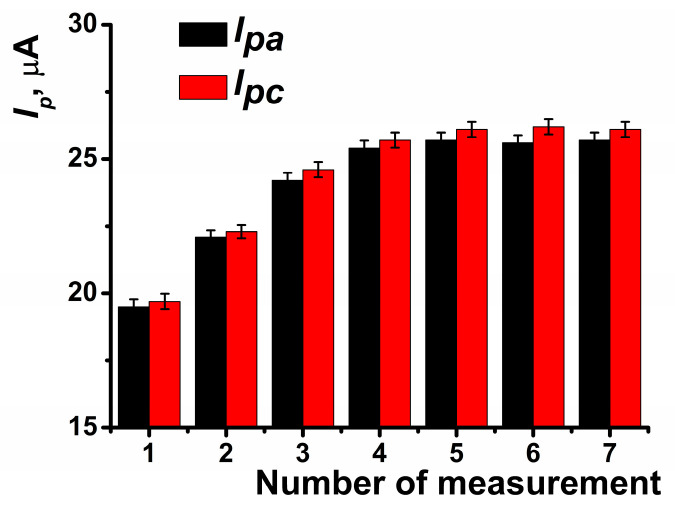
Anodic and cathodic peak currents recorded on SPCE modified with PB and CB depending on the number of voltammetric measurements performed. Average of the results obtained using five SPCE prepared from the same reagent set. Measurements were performed in 0.01 M phosphate buffer solution + 0.1 KCl, pH = 7.0. Scan rate 0.1 V∙s^−1^.

The pre-application of CB significantly enhanced the peak current by approximately 30% (Figure 3). In subsequent measurements, the peak current continued to increase until the fourth measurement, after which the coating stabilized (Figure 4).

**Figure 5:**
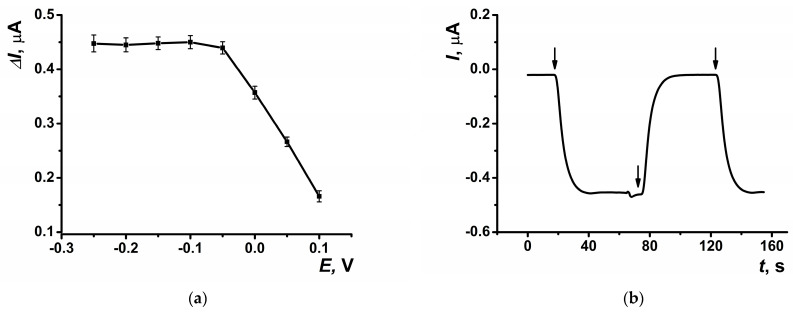
(**a**) The dependence of the maximal current shift, and (**b**) dynamic response of the sensor at E = −0.05 V on SPCE modified with CB and PB in the presence of hydrogen peroxide (10 μM). Arrows indicate switching the flows from phosphate buffer solution to hydrogen peroxide solution and back. Average ± S.D. for five SPCEs prepared from the same reagent set. Amperometric measurements were performed at a flow rate of 0.2 mL∙min^−1^ in 0.01 M phosphate buffer solution + 0.1 M KCl, pH = 8.

**Figure 6:**
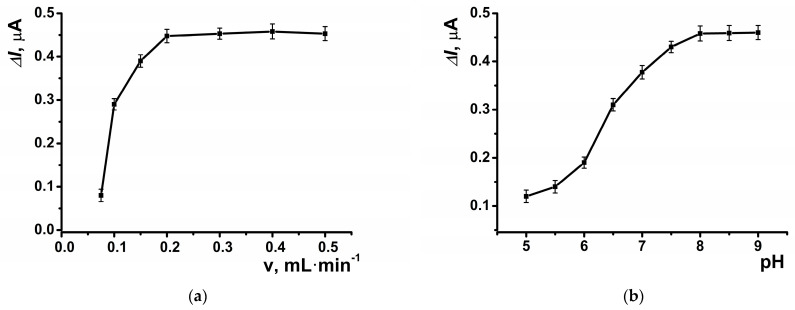
The dependence of the maximal current shift for SPCE modified with CB and PB in the presence of hydrogen peroxide (10 μM) at E = –0.05 V on (**a**) the flow rate, and (**b**) pH of the buffer solution. Average ± S.D. for five SPCEs prepared from the same reagent set. Amperometric measurements were performed at a flow rate of 0.2 mL·min^−1^ in 0.01 M phosphate buffer solution + 0.1 M KCl, pH = 8.

The highest response to hydrogen peroxide in amperometric mode within the flow-through system was observed at a potential of –0.05 V, which was selected for subsequent experiments (Figure 5a). The dynamic response of the amperometric sensor is presented in Figure 5b. At the selected potential, the background signal of the modified coating was negligible, measuring approximately 20 nA. The highest amperometric response was observed at a flow rate of 0.2 mL∙min^−1^ (Figure 6a) and pH = 8 (Figure 6b).

**Figure 7:**
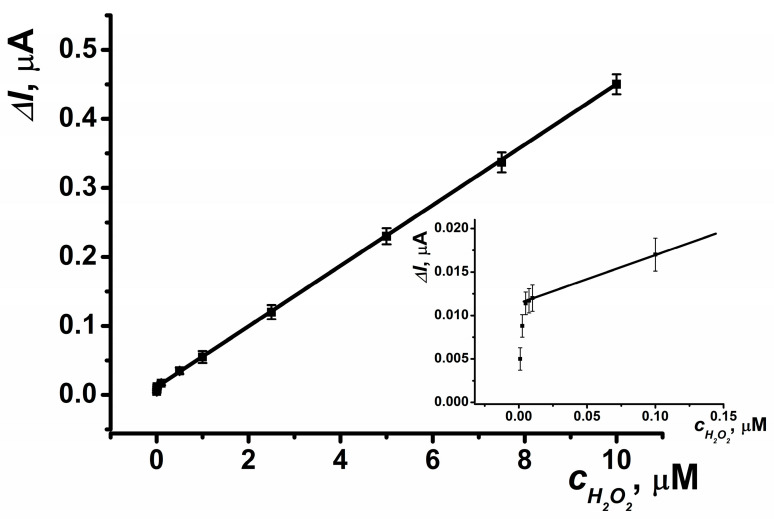
Calibration curve of hydrogen peroxide determination for SPCE modified with CB and PB. Average ± S.D. for five SPCEs prepared from the same reagent set. Amperometric measurements were performed at E = –0.05 V, a flow rate of 0.2 mL·min^−1^ in 0.01 M phosphate buffer solution + 0.1 M KCl, pH = 8.

The electrode demonstrated a linear determination range for hydrogen peroxide spanning from 5 nM to 10 μM (Figure 7). The parameters of the linear regression equation for this coating were as follows: *ΔI*, μA = (12.21 ± 0.32) + (43.59 ± 0.18) × (*c*, nM), R^2^ = 0.9998. This system enables up to 40 measurements per hour using the same electrode.

### 3.4. Biosensor System for Uric Acid Determination

For the determination of uric acid, the inner wall of the reactor was modified with the enzyme uricase. In the presence of uric acid and dissolved oxygen, hydrogen peroxide is produced via the enzymatic reaction, which is subsequently detected through cathodic reduction at the SPCE located beneath the reactor within the flow-through cell.

**Figure 8:**
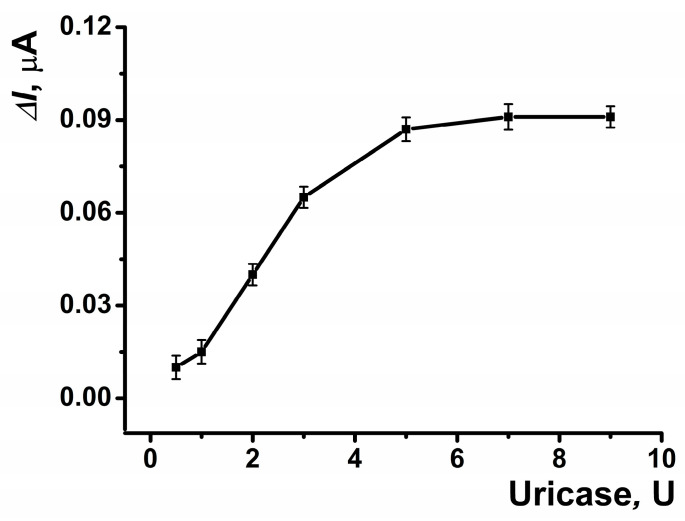
The dependences of the maximal current shift for SPCE modified with CB and PB in the presence of uric acid (5 μM) on the amount of the enzyme on the inner surface of the reactor. Average ± S.D. for five SPCEs prepared from the same reagent set. Amperometric measurements were performed at E = –0.05 V, a flow rate of 0.2 mL·min^−1^ in 0.01 M phosphate buffer solution + 0.1 M KCl, pH = 8.

The reactor of the electrochemical cell was fabricated from PLA, utilizing its terminal carboxyl groups for carbodiimide binding to immobilize the enzyme on the inner surface of the reactor (Scheme 1). The highest response to uric acid was observed with 5 U of enzyme immobilized on the reactor surface (Figure 8).

**Figure 9:**
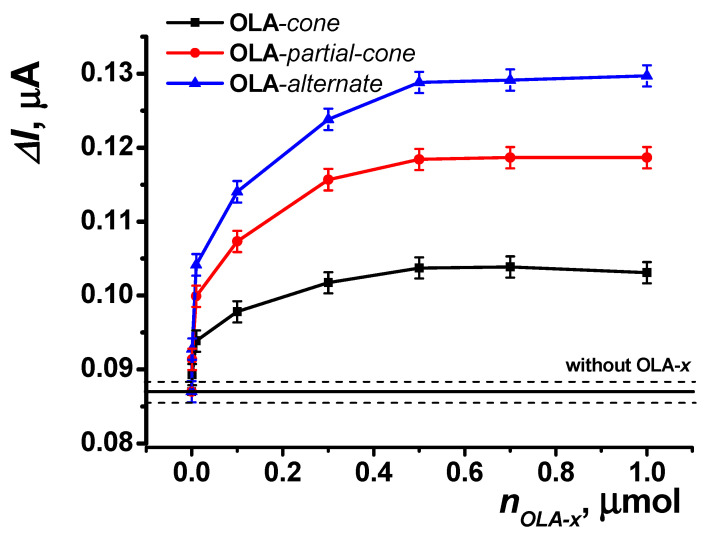
Dependence of the maximal current shift for SPCE modified with CB and PB in the presence of uric acid (5 μM) on the amount of functionalized oligo (lactic acid) on the reactor surface (5 U uricase immobilized on the reactor surface). Data represent the average ± S.D. for five SPCEs prepared from the same reagent set. The horizontal solid line and dashed lines indicate the response level ± S.D. for the reactor surface without OLA modification. Amperometric measurements were performed at E = –0.05 V, a flow rate of 0.2 mL·min^−1^ in 0.01 M phosphate buffer solution + 0.1 M KCl, pH = 8.

To enhance the efficiency of enzyme immobilization, an acetone solution of thiacalixarene-functionalized oligo (lactic acids) was applied to the reactor surface. The recorded amperometric response increased with the addition of increasing amounts of the functionalized polymers used, reaching a saturation point at 0.8 μmol on the reactor surface (Figure 9).

This effect is further enhanced across the series of stereoisomers (OLA-cone < OLA-paco < OLA-alt), as observed in the swelling experiments (see Section 3.2). The enhancement is attributed to steric pre-organization within the structures of the thiacalixarene-functionalized oligo (lactic acids). For the most branched OLA-alt, the spatial arrangement ensures that a greater number of carboxyl groups on the oligo (lactic acid) remain accessible for binding, irrespective of the polymer’s distribution on the reactor surface, compared to other conformations of *p-tert*-butylthiacalix[4]arene.

**Figure 10:**
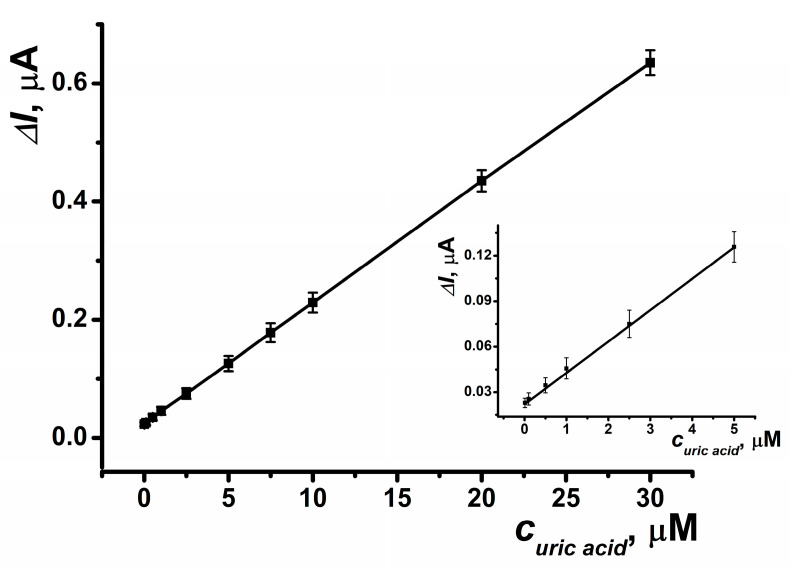
Calibration curve of uric acid determination obtained in a SPCE reactor modified with OLA-alt (0.5 μmol), uricase (5 U), CB and PB. Average ± S.D. for five SPCEs prepared from the same reagent set. Amperometric measurements were performed at E = –0.05 V, a flow rate of 0.2 mL·min^−1^ in 0.01 M phosphate buffer solution + 0.1 M KCl, pH = 8.

The resulting biosensor system, which incorporates an SPCE modified with CB and PB, and a reactor modified with OLA-alt (0.5 μmol) and uricase (5 U), enables the determination of uric acid in the range of 10 nM to 30 μM with a limit of detection (LOD) of 5 nM, calculated using the S/N = 3 criterion (Figure 10). The parameters of the linear regression equation for this setup are *ΔI*, μA = (23.46 ± 0.29) + (20.49 ± 0.08) × (*c*, nM), R^2^ = 0.9998. This biosensor system allows for up to 20 measurements per hour using the same electrode. The immobilized enzyme retained its activity under continuous flow conditions for at least 8 h. Additionally, when stored in dry conditions, 50% of its activity was retained six months post-manufacture.

The analytical performance of uric acid determination using the developed biosensor, as summarized in Table 2, is comparable to or exceeds that of previously reported electrochemical sensors.

Notably, the application of such sensors in medical diagnostics often involves measuring higher levels of uric acid, enabling multiple dilutions of biological samples to minimize the interfering effects of matrix components. A significant advantage of cathodic current measurement is the minimal influence of oxidizable compounds on the biosensor signal. The following potential interferents were tested, with their maximum concentrations that showed no effect indicated in parentheses: glucose (5 mM), dopamine (1 mM), and ascorbic acid (0.1 mM). The biosensor system was further evaluated using artificial urine and synthetic blood plasma samples. For artificial urine, the recovery rate was 121% for the undiluted sample and 101% for a tenfold dilution with phosphate buffer solution. In synthetic blood plasma, the recovery was 134% for the undiluted sample and 102% for a hundredfold dilution with phosphate buffer solution. Given the normal physiological levels of uric acid in biological fluids, the developed biosensor system provides accurate determination of this biomarker, sufficient for the early diagnosis of related diseases.

The results of uric acid determination in artificial urine and synthetic blood plasma are presented in Table 3.

## 4. Conclusions

The developed 3D-printed flow-through electrochemical cell was designed based on principles of miniaturization and ease of assembly. Unlike most devices requiring additional fasteners for assembly, this cell is secured with a single rotary motion due to the threaded design of the upper and bottom parts. The pressure applied by the upper reactor part is sufficient to ensure the tightness of the internal space between the electrode and the reactor, eliminating the need for additional seals and simplifying the design further.

The reactor was fabricated from PLA, which was modified with thiacalixarene-functionalized oligo (lactic acids). This study demonstrated that poly(lactic acids) modified with *p-tert*-butylthiacalix[4]arene derivatives exhibited greater resistance to swelling and hydrolytic degradation compared to unmodified oligo (lactic acid). This effect was enhanced in the series of conformation stereoisomers: OLA-cone < OLA-paco < OLA-alt. The stability of the studied oligo (lactic acids) varied with the medium’s acidity (9.2 < 4.0 < 6.9), reaching a maximum at neutral pH values near the physiological pH of blood. Modifying the reactor material increased the immobilization efficiency of the uricase enzyme, resulting in an enhanced uric acid signal while protecting the reactor from swelling and hydrolytic degradation. Among the modifications studied, poly(lactic acids) functionalized with thiacalixarene in the *1,3-alternate* conformation produced the best results.

The working electrode for hydrogen peroxide detection was modified with carbon black and Prussian blue. Carbon black ensured the coating’s stability in the flow, while Prussian blue decreased the potential for uric acid determination to –0.05 V, a marked improvement compared to previously reported systems [31].

The performance of the flow-through biosensor was evaluated using uric acid determination. Uric acid, an important biomarker for various pathologies, can thus be monitored in real time. The developed biosensor allowed the determination of 10 nM to 30 μM uric acid with LOD of 5 nM, including in samples of artificial urine and synthetic blood plasma. The biosensor’s performance was comparable to or better than its analogs. Using cathodic currents avoided interference from most oxidizable substances, enhancing the system’s reliability. These factors highlight the potential of the developed flow-through biosensor system for low-cost metabolite monitoring in biological fluids.

The system’s adaptability allows for easy modification with other enzymes or conversion into a point-of-care testing format. Additionally, the PLA material used in the reactor can be functionalized with other compounds for diverse applications, further expanding the immobilization possibilities on the reactor surface. The low flow rates, small reactor volume, and cost-effective manufacturing process enable this system to compete with more complex microfluidic devices or implantable biosensors.

## Figures and Tables

**Table 1:** Degree of swelling and weight loss of oligo (lactic acid) samples immersed in buffer solutions (pH 4.0, 50 mM potassium phthalate; pH 6.9, 50 mM potassium dihydrophosphate; pH 9.2, 10 mM sodium borate).

Sample	pH 4.0 24 h		pH 6.9 24/168 h		pH 9.2 4 h	
	Degree of Swelling (%)	Weight Loss (%)	Degree of Swelling (%)	Weight Loss (%)	Degree of Swelling (%)	Weight Loss (%)
OLA	25	13	20/– *	3/– *	75	26
OLA-cone	17	10	15/– *	<0.5/– *	35	8
OLA-paco	16	9	12/292	<0.5/30	20	9
OLA-alt	6	4	<0.5/73	<0.5/21	5	4

* “–” indicates complete destruction of the sample.

**Table 2:** Comparison of the electrochemical characteristics of the developed biosensor with other electrochemical sensors for uric acid determination described in the literature.

Electrode/Modifier	Detection Mode	Concentration Range, M	LOD, M	Ref.
Glassy carbon electrode, reduced graphene oxide/polypyrrole-platinum nanocomposite	Differential pulse voltammetry	1 × 10^−4^–3.5 × 10^−4^	1.6 × 10^−7^	[72]
Glassy carbon electrode, Co-N/Zn/nanoporous carbon, zeolite-like imidazolate framework	Differential pulse voltammetry	1 × 10^−7^–1.47 × 10^−5^	1 × 10^−7^	[73]
SPCE, polycaprolactone, polyethylene imine, uricase, quantum dots	Differential pulse voltammetry	5 × 10^−6^–5.2 × 10^−5^	3.96 × 10^−8^	[74]
Laser-induced graphene electrode, bovine serum albumin, TWEEN-20	Differential pulse voltammetry	2 × 10^−5^–1 × 10^−3^	2.1 × 10^−6^	[75]
Uricase on reactor, PLA modified by *p-tert*-butylthiacalix[4]arene in *1,3-alternate* conformation, SPCE, CB, PB	Differential pulse voltammetry	1 × 10^−8^–3 × 10^−5^	7 × 10^−9^	This work

**Table 3:** The results of uric acid determination and recovery measurements in spiked samples of artificial urine and artificial blood plasma using the developed biosensor system.

Measurement Medium	Uric Acid, μM		Sr	Recovery, %
	Spiked	Found		
Undiluted artificial urine	10	12.1 ± 0.2	0.02	121
3 times diluted artificial urine		10.7 ± 0.1	0.01	107
10 times diluted artificial urine		10.1 ± 0.1	0.01	101
Undiluted synthetic blood plasma	10	13.4 ± 0.2	0.02	134
10 times diluted synthetic blood plasma		11.2 ± 0.2	0.02	112
100 times diluted synthetic blood plasma		10.1 ± 0.1	0.01	102

## Data Availability

The datasets presented in this article are not readily available because the data are part of an ongoing study.
